# A Methodology for Semantic Enrichment of Cultural Heritage Images Using Artificial Intelligence Technologies

**DOI:** 10.3390/jimaging7080121

**Published:** 2021-07-22

**Authors:** Yalemisew Abgaz, Renato Rocha Souza, Japesh Methuku, Gerda Koch, Amelie Dorn

**Affiliations:** 1 ADAPT Centre, School of Computing, Dublin City University, Glasnevin Campus, Dublin 9, Dublin, Ireland; Japesh.Methuku@adaptcentre.ie; 2 Austrian Centre for Digital Humanities and Cultural Heritage (ACDH-CH OeAW), Austrian Academy of Sciences, 1010 Vienna, Austria; renato.souza@oeaw.ac.at (R.R.S.); Amelie.Dorn@oeaw.ac.at (A.D.); 3 AIT Angewandte Informationstechnik Forschungsgesellschaft mbH, Europeana Local AT, Klosterwiesgasse 32, 8010 Graz, Austria; Kochg@europeana-local.at

**Keywords:** cultural images, cultural heritage, artificial intelligence, computer vision, semantic enrichment, image analysis, digital humanities, ontologies, deep learning

## Abstract

Cultural heritage images are among the primary media for communicating and preserving the cultural values of a society. The images represent concrete and abstract content and symbolise the social, economic, political, and cultural values of the society. However, an enormous amount of such values embedded in the images is left unexploited partly due to the absence of methodological and technical solutions to capture, represent, and exploit the latent information. With the emergence of new technologies and availability of cultural heritage images in digital formats, the methodology followed to semantically enrich and utilise such resources become a vital factor in supporting users need. This paper presents a methodology proposed to unearth the cultural information communicated via cultural digital images by applying Artificial Intelligence (AI) technologies (such as Computer Vision (CV) and semantic web technologies). To this end, the paper presents a methodology that enables efficient analysis and enrichment of a large collection of cultural images covering all the major phases and tasks. The proposed method is applied and tested using a case study on cultural image collections from the Europeana platform. The paper further presents the analysis of the case study, the challenges, the lessons learned, and promising future research areas on the topic.

## 1. Introduction

The digitisation of cultural heritage resources has opened a new way of sharing and utilising information that was previously offline. Many Galleries, Libraries, Archives, and Museums (GLAMS) transform tangible and intangible heritage by converting the physical resources into digital images, audios, videos, simulation models, and virtual realities [1]. As part of the effort, cultural images that represent the culture and history of societies become available in digital formats on the semantic web [2,3,4]. UNESCO defines cultural heritage to encompass tangible heritage including movable (paintings, sculptures, coins, and manuscripts), immovable (monuments, archaeological sites), and underwater cultural heritage; and intangible heritage covering oral traditions, performing arts, and rituals (http://www.unesco.org/new/en/culture/themes/illicit-trafficking-of-cultural-property/unesco-database-of-national-cultural-heritage-laws/frequently-asked-questions/definition-of-the-cultural-heritage/ (accessed on 15 April 2021)). Cultural heritage images include paintings, photographs, drawings, and sketches that represent the culture and/or history of a particular society or country [4]. Although cultural images are available embedded on a physical medium, the massive digitisation process makes them accessible in a digital intangible format. In this paper, digital cultural heritage images (we refer to them as cultural images now onwards) represent a selection of digital images that reflect the culture of a society in the past and present. Since culture encompasses a wider range of human aspects, it is difficult to fully understand and cover all these aspects. This paper focuses on cultural images that are related to edible food.

Despite the growing number of cultural images, the availability and the maturity of methods and tools to systematically exploit the content of the images in a structured and meaningful way is still at its early stage [5,6]. Solutions that work well in other domains (such as medical imaging) were not exploited until recently. Elsewhere, digital images are widely represented by metadata related to the creators, creation time, title, and short descriptions of the images. However, these representations lack contextual information about the cultural content of the images. For this reason, the most valuable information embedded in the images is not explicitly annotated and utilised.

In the light of recent advancements in AI, there are now greater opportunities for digital humanities to apply sophisticated AI solutions to enrich and exploit cultural images [7]. Natural language processing [8], image classification [9,10], Computer Vision (CV) [11], and Virtual Reality (VR) are some of the areas that are gaining strong momentum and fast adoption in digital humanities research. CV in particular has been used to analyse cultural heritage collections including architectures, buildings, and other cultural artefacts. The analysis includes object detection, classification, reconstruction, and semantic annotation. Ontologies [12] have been proven to be crucial for semantic enrichment of cultural images. Ontologies are used to consistently represent resources to be understood and interpreted uniformly by humans and machines [13] in the Linked Open Data (LOD) space [14].

Despite the availability of technological solutions, the digital humanities domain has not yet exploited the full benefits due to the lack of an end-to-end methodology that supports the transformation of cultural images from mere digital entities to useful resources for supporting scientific research. Unless addressed methodologically, the use of existing technologies for searching, analysing, and annotating cultural images with such usable content can be breathtakingly time-consuming. Moreover, the absence of ground truth which would normally serve as a basis for the development and evaluation of AI solutions is lacking. An interpretation template for both concrete and abstract concepts of cultural images is missing [15]. Thus, a combination of manual and automatic annotation is widely proposed to semantically enrich cultural images.

To date, CV and image analysis technologies focus on detecting concrete objects in the images [15]. Although the technologies serve well for object detection, they are in their early stage generating usable annotations for abstract and highly subjective aspects [16] of cultural images. Moreover, most of the CV technologies are trained using images that emerged from domains that have sufficient and quality data for the training and evaluation of such systems [17].

This paper presents a three-phase methodology for semantic enrichment of cultural images using AI technologies. Our methodology begins with the preparation phase which enables us to understand the domain, acquire the target resources including the images, ontologies, and vocabularies. The second phase presents tools and techniques for analysing and annotating the content, training and evaluating the CV models, whereas phase three focuses on deployment, exploration, and integration of active learning components in the system. Our methodology follows a continuous and iterative development within and across the phases. The proposed methodology is developed in the framework of the ChIA project (ChIA—accessing and analysing cultural images with new technologies) [18] where AI solutions are applied to solve problems in the digital humanities domain. The contribution of the paper includes:

- An end-to-end methodology and case study for semantic enrichment of cultural images.
- A technique for building and exploiting CV tools for digital humanities by employing iterative annotation of sample images by experts.
- A vocabulary for enriching cultural images in general and images related to food and drink in particular.
- A benchmarking data set which could serve as a ground truth for future research.
- A discussion of the lessons, challenges, and future directions.

The remaining sections of the paper are organised as follows. Section 2 introduces the complex aspects of cultural images and how they are represented and analysed using CV and semantic web technologies. Section 3 outlines our proposed methodology which is organised into three phases: preparation, analysis, and integration and exploitation. In Section 4, we present a case study where our proposed methodology is applied to cultural images collected from Europeana and Europeana-local Austria. Following the findings of the case study, the discussion of the results is presented in Section 5. Finally, recommendations and future work are presented in Section 6.

## 2. State of the Art

Until recently, the focus of digital humanities research was on the conversion of resources into a digital representation and publishing mainly in platforms supported by the respective institutions [7,19]. However, the emergence of new image processing technologies, deep learning, natural language processing, and semantic web technologies provides new opportunities to enhance the organisation, interlinking and exploitation of cultural heritage images. In this section, we summarise the advancements in these areas.

### 2.1. Computer Vision in Digital Humanities

Computer Vision applications such as image recognition, object detection, and classification using large-scale digital images have gained significant traction in digital humanities research. In this section, we review the advancements in Convolutional Neural Network (CNN) in light of their application in the digital humanities domain.

#### Convolutional Neural Networks

CNN comprises different layers like convolution, pooling, and activation that help in analyzing the patterns in an image. The convolution layers form a core building block of a CNN where each layer consists of a set of K learnable filters, each filter having a width and height. The output of each convolution operation produces an activation map which is a 2-dimensional output. The images are represented by pixels and mathematical operations are used by CNN for analyzing the patterns embedded within the images. CNNs are built using a sequence of convolution, pooling and non-linearity layers where convolution is used to extract spatial features and pooling layers are used to reduce the spatial dimensions of the image.

ImageNet is a benchmark data set having around 15 million labelled images that represent 22,000 categories. ImageNet Large Scale Visual Recognition Challenge (ILSVRC) uses around 1.2 million images for training, 50,000 images for validation and 100,000 images for testing. CNN architectures are designed to classify the images for ILSVRC and the architectures have evolved. LeNet5 [20] was one of the simplest CNN architectures having two convolution and three fully connected layers. The architecture in which the convolution and pooling layers were stacked in LeNet5 turned a baseline for other CNN models. AlexNet [21] was the next benchmark CNN architecture that was a much deeper and wider version of LeNet5 and could learn much more complex objects and used Rectified Linear Units (ReLU) as non-linearities. The architecture also saw the use of dropout regularisation which is a technique in deep learning to reduce the effect of overfitting (models’ ability to generalize on unseen images is suppressed) and also data augmentation techniques which allows the CNN model to visualize the images by applying different properties like translation, reflections, and patch extractions. The data augmentation technique is particularly useful when there is minimum availability of images for training a CNN model as it introduces new variations into the data set. AlexNet has eight layers with five convolutional and three fully connected layers.

What changed between LeNet5 and AlexNet is the number of layers stacked to design a CNN architecture. With the increase in depth of the layers in a CNN, there was an improved chance of learning complex patterns and representations, and these patterns resulted in more complex architectures going much deeper and with more trainable parameters. VGG (Visual Geometry Group) network [22] was designed and developed by the researchers at Oxford University which has thirteen convolutional and three fully-connected layers with ReLU as non-linearity. There are two variants of the VGG network, VGG16, and VGG19 and use smaller 3 × 3 filters in each convolutional filter. These multiple smaller filters can emulate the effect of larger receptive fields to represent complex features. However, a network with such large depth also makes the model bigger, and VGG network has 138 million trainable parameters.

ResNet50 [23] was trained on ImageNet data set with a 152 layer deep convolutional neural network, which is eight times deeper than the VGG network. An ensemble of the residual networks achieved a 3.57% error on the ImageNet test set. The experiments were conducted to understand the use of residual learning and shortcut connections for improving the generalizability of the model. Convolution and identity blocks form the basic building block of ResNet50 and this CNN model has 26 M parameters.

Inception_V3 [24] is a variant in the inception family of pre-trained convolutional neural networks, the architecture of which is reviewed by rethinking the inception architecture to realize computational efficiency and fewer parameters. The Inception_V3 architecture is composed of factorized convolutions where the aim is to reduce the number of connections/parameters without decreasing the performance/efficiency of the neural network. The idea behind factorized convolutions is to replace a convolution of a larger receptive field with smaller size convolutions. For example, one 5 × 5 convolution layer can be decomposed into two 3 × 3 convolution layers, which further reduces the number of parameters. Furthermore, a kind of dropout regularization technique, label smoothing is used to prevent the logits from taking large values. Label smoothing also helps in preventing the CNN model from overfitting.

With the evolution of CNN architectures, there has been a lot of research to reduce the complexity of the model by making the models much deeper. In total, 1 × 1 (pointwise) convolutions were adapted in the models using which the features across the feature maps could be spatially combined with the effective use of very few parameters. Depthwise convolutions is one such idea that comprises two convolution operations, spatial convolution followed by pointwise convolutions. This made the CNN networks lighter and faster due to fewer trainable parameters and fewer FLOPs (floating-point operations). Xception [25] is an improvement and an extension of the inception family of CNN architectures with few architectural changes and effective as ResNet50 and Inception_V3. In Xception, depthwise separable convolutions have replaced the inception modules. There is a performance improvement in Xception due to the more efficient use of model parameters. The pointwise convolutions are followed by depthwise convolutions, unlike the inception network. The Xception architecture is divided into three flows, entry flow, middle flow, and exit flow. The data is passed through the entry flow, and the middle flow is repeated eight times and then the data is passed through the exit flow.

Machine Learning has been used in the space of digital humanities to classify images belonging to cultural heritage in [10], where there is a comparison of different approaches like multilayer perceptron, k-nearest neighbour, and CNNs. The classification was based on concrete concepts that are well defined and the patterns within the image attribute to a particular class/category. We aim to investigate and analyze how CNNs can be used for abstract concepts (Section 3 and Section 4). We have used three pre-trained models, Inception_V3, ResNet50, and Xception to classify the images based on abstract concepts.

There are a few shortcomings of computer vision and deep learning algorithms used for the classification of images. First, they are mostly trained to support general-purpose applications which might not be effective for very specific domains. Second, the models are trained to recognise well-known concrete objects, shapes, colours, etc. However, in the cultural images, the interest encompasses the identification of abstract concepts represented in the cultural images such as formality, appealingness, etc. Another shortcoming is that the techniques used in designing these models work mathematically well, but are is often claimed as being black boxes where there are no set of rules for maximizing the results. This is deeply concerning because it minimizes the opportunity to verify the decision-making process while working towards the objectives.

### 2.2. Semantic Web Technologies

The semantic web refers to an extension of the World Wide Web with a goal of encoding semantics to the data on the web to facilitate the interlinking of web resources and to support machine-readable format. The semantic web uses technologies such as Resource Description Framework (RDF (https://www.w3.org/TR/owl-features/ (accessed on 13 July 2021))) and Web Ontology Language (OWL (https://www.w3.org/TR/rdf-syntax-grammar/ (accessed on 13 July 2021))) to facilitate encoding and processing meaning for the consumption of human and computer agents [26]. Semantic web technologies benefited from the development of large repositories (DBpedia [27], Europeana, and swissbib (https://data.swissbib.ch/ (accessed on 10 May 2021))), multilingual and inter-disciplinary vocabularies (BabelNet [28], WordNet [29]), specialised ontologies (CIDOC-CRM [30], EDM [31]), and the LOD initiatives. Such repositories not only provide the required vocabularies to enrich cultural images but also enable semantic interlinking of the resources and creating links that can be exploited by both human and artificial intelligent agents.

Previous research exploits the semantic web technologies in different forms. An ontology model for narrative image annotation has been developed to annotate images in the field of cultural heritage [14]. The authors developed an ontology model and a tool to semantically annotate narrative images. However, the image annotation is done manually being supported by the tool. Marcia [13] presented a review of semantic enrichment efforts in Libraries, Archives and Museums (LAM). The application of semantic enrichment in LAM includes the development of ontologies and semantic annotation of structured and unstructured digital resources. Although this is a review paper, it identified several semantic enrichment projects using ontologies, linked data and SPARQL queries to organise, search and retrieve digital resources. Another effort towards accessing historical and musical linked data is proposed in [32]. A web-based thin middleware that facilitates the use of SPARQL queries to access digital humanities linked data sets on the web is proposed. This paper presents a prototypical tool that allows the use of API-based access to enable users to interact with the linked data without using SPARQL queries. Although this paper focuses on the exploitation of semantic data sets, it also demonstrates the gap in the digital humanities domain.

Currently, the major challenge in this area includes the coverage of specialised ontologies that represent domain-specific concepts to interpret and understand consistently [33]. Most of the ontologies do not always cater to the needs of new applications. In this regard, although there is a continuous development of domain-specific ontologies and vocabularies representing major cultural aspects, it requires a substantial effort to integrate the ontologies/vocabularies to make a significant impact. Another observed gap in the literature is the slow adoption of the application of CV models to automatically detect and annotate abstract cultural aspects. CV models are capable of detecting objects and generating labels that can be fed with standard ontologies to generate labels represented by unique URIs to ensure consistent representation of the images.

## 3. Methodology for Enhancing the Visibility of Cultural Images

**Figure 1:**
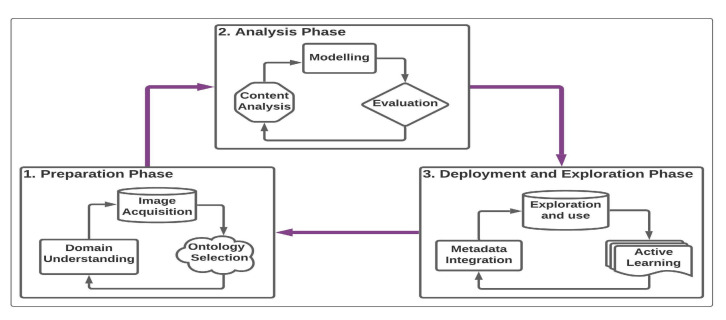
A three-phase-methodology for semantic enrichment of cultural images.

The proposed methodology (Refer to Figure 1) is organised into preparation, analysis, and integration and exploitation phases. The first phase focuses on acquiring, understanding, and representing the target domain and its related data. The second phase deals with the extraction of the content of the images using CV models and the last phase focuses on the integration and exploitation of the results of previous phases to provide rich information. The methodology follows an iterative and continuous improvement in each of the phases.

### 3.1. Phase-1: Preparation Phase

Some of the challenges faced in the semantic enrichment of cultural images include the complexity and diversity of the collection [34]. Most often, there is no one-fits-all solution that serves well all kinds of collections. Thus, a preparation phase that defines the domain of interest, acquiring representative data, and selecting the appropriate vocabulary is crucial to any digitisation and semantic enrichment project.

#### 3.1.1. Domain Understanding

Cultural images represent tangible artefacts such as buildings, food, cloth, machinery, and intangible artefacts such as festivities, language, music, and others [4]. Understanding the domain and defining the boundaries of the collection at the very early stage enables the selection and filtering of the target images and potential domain-specific ontologies. Given a large collection of digital images, applying semantic enrichment on the full collection in one step will result in a broader but shallow semantic annotation, whereas, focusing on a particular topic enables a deeper and rich semantic representation.

Thus, the first step in this process is understanding the collection and defining the topics that will be included in the semantic enrichment process. Focusing on the topic, where the target images deal with a particular subject such as food, drink, farming, and wedding. The additional dimension of the domain could include temporal information such as ancient, medieval, or modern eras or artefacts from specific seasons. The type of images including paintings, drawings, sketches, photographs could be used as additional criteria to defining the domain and set the boundaries.

Although several GLAMs focus on specific subjects, and times, aggregation platforms such as Europeana [34] expose very wide and diverse cultural images which pose additional challenges. In such situations, this particular step becomes very crucial for narrowing down the domain.

#### 3.1.2. Image Acquisition

This step involves the process of acquiring cultural images that are relevant to the selected domain in a digitised format. The image acquisition process could be specific to collections that are already available on existing platforms or new ones. This step becomes time-consuming particularly when narrow subject areas are selected. Even, with the support of efficient search and retrieval tools, current platforms often do not provide accurate and reliable results due to the quality of the associated metadata and the lack of rich semantics. This step further requires the allocation of significant manpower to spare on manual inspection and filtering. Image acquisition is done by domain experts in the topic area or using specialised tools that facilitate the selection of images relevant to the domain.

#### 3.1.3. Ontology Selection

Another crucial step in the preparation phase is the selection of suitable and rich semantics. Ontologies provide the semantic meaning and representation of concepts of a domain [12]. Although generic ontologies representing widely applicable concepts can be used, the main focus of this step is the identification and selection/composition of ontologies that represent the concepts of interest of the selected domain both in its breadth and depth.

The selection of ontologies that are suitable for the semantic annotation of cultural images is often guided by the task at hand. There are several widely used criteria for selecting the right ontologies for specific tasks [35]. Once candidate ontologies are identified, often the decision would be selecting one or more of the identified ontologies or deciding to create a new ontology from scratch based on functional and non-functional requirements. Some of the functional requirements to determine the availability and suitability of ontologies include the coverage of the target concepts, the number of relationships captured and represented, and the expressiveness of the ontology. The non-functional requirements include a continuous maintenance and sustainability, availability for free re-use, compatibility with the standard (e.g., ISO 25964 norm) and its support for linked open data usage (similar to SKOS-Format).

### 3.2. Phase-2: Analysis Phase

This phase focuses on the automatic extraction of the content of the images. This analysis is not a trivial task, particularly identifying abstract and subtle concepts from cultural images is often difficult and subjective. However, we believe that a systematic approach that integrates expert input and active learning methods can ensure the extraction of concepts at least to the level of agreement observed between experts.

#### 3.2.1. Analysis of the Content of Images

A semantic analysis of the target images preferably by several domain experts not only provides a useful, and often an accurate representation of the content of the images, but also exhibits the level of agreement, detail, and difficulty that involve in the semantic enrichment process. Each image is analysed by experts and annotated using the selected concepts. The annotation process of concrete concepts (e.g., fruit, animal, vehicle, etc.) usually shows a higher inter-annotator agreement whereas annotation of abstract images exhibits lower (sometimes random) agreement. To avoid the subjectivity of the annotation, the analysis also includes the percentage of agreement exhibited between the annotators by including the statistics as a probability along with the annotated concepts for each of the images.

Processing the inter-annotator agreement between the annotators and understanding the nature and level of agreement in the annotation process of the selected domain provides crucial information in setting a benchmark for the envisioned automated annotation tool. Where humans display a higher level of agreement, automated systems are also expected to perform to the same level as human performance. Whereas, when the level of agreement between human annotators becomes low, the implication is that there is a huge subjectivity in the task which also requires to be captured in the automated systems. Due to the subjectivity involved in labelling cultural images with abstract concepts, instead of generating deterministic annotations, the area would benefit from fuzzy annotation [36] of images representing some level of uncertainty [37].

#### 3.2.2. Preparation of Training Data

For most of the tasks involving the preparation of training data for CV experiments, a large collection of training data is required. However, with the emergence of pre-trained models, this can be leveraged by reusing those pre-trained models in combination with a small set of new annotations focusing on particular features of the images. This leverages a significant portion of the task of collecting training data. However, for tasks that require domain-specific and in-depth analysis of the contents of the images, finding sufficient training data is still a major problem. What works for a general semantic annotation task does not often work for domain-specific annotations due to the requirement of domain experts. Thus, the training data is often restricted to a few thousands of images. Methods to tackle the problem include exploiting available domain experts to train annotators to achieve a better understanding of the domain, engaging experts in a more creative approach, or relying on existing metadata and NLP tools to see if any pattern from the annotations of domain experts could be learned and generalised.

Existing CV models allow the use of previously trained models with a different set of images to be used to train new and unseen images and categories. Although this improves the learning rate of the algorithms, any successful CV tool still requires a large data set for training, validation, and testing.

#### 3.2.3. Training and Selection of Best Performing Model

Recently, several computer-based image annotation methods became available. Among these CNN methods are gaining significant popularity [38,39]. The major considerations in selecting these CNN methods depend on their accuracy in generating results that are similar or superior to the accuracy achieved by human experts. In this phase, researchers train several models to select either the best performing one or ensemble two or more models to achieve higher performance. The selection is guided by the accuracy of the models during the training, validation, and testing phases.

The next step after the selection of the best-performing model is to use the selected model to label unseen images to obtain new annotations. These annotations will further include the predicted probability. The model uses a confidence level (0–100%) and this confidence level will be used to represent the confidence of the predicted annotation. The resulting data from the annotation will generate a list of annotation triples for each of the target images in a form of a CSV file (Example: Annotation.csv). A generic annotation format as a file is presented in Table 1.

### 3.3. Phase-3: Integration and Exploitation Phase

One of the important factors in semantic enrichment is the integration of new semantic annotations into the existing semantic repositories. The integration process introduces new metadata to further describe the target resource. In systems that already have semantic repositories, the integration considers the legacy system and tries to integrate the new metadata in the legacy system without breaking the consistency and the validity of the data.

For resources that do not have a legacy semantic repository, the task involves creating the metadata repository, which further includes the selection, implementation, and deployment of a semantic repository. However, the selection and deployment of the repositories are out of the scope of this research paper. Further reading on the topic is available in [40,41,42,43].

#### 3.3.1. Integration of Results

The integration of large-scale semantic annotations generated by the annotation models involves the transformation of the generated annotation into a semantic representation. This process involves the use of subject-predicate-object triples where the subject represents the unique identifier (URL) of the image. The predicate represents the relationship between the subject and the object. The object is the predicted label that is generated by the model. Where there are several annotations available for a target image, an s-p-o triple will be generated for each of the annotations. A mapping of the annotation file into RDF format is carried out by using R2RML mapping [44]. A typical R2RML mapping converts the input annotation into its equivalent RDF file using the following R2RML mapping (https://github.com/yalemisewAbgaz/ChIA_Semantic_Annotation.git (accessed on 15 July 2021)).

@prefix <list all your prefixes here>.
 
<#TripleMap1> a rr:TriplesMap ;
rr:logicalTable [rr:tableName "PREDICTIONS"];
rr:subjectMap[rr:template "https://www.europeana.eu/en/item/{IMAGE_NAME}";
rr:class edm:webResource; ];
rr:predicateObjectMap[rr:predicate dc:description;
rr:predicate rdfs:comment;
rr:objectMap[rr:column "LABEL"; ];];
rr:predicateObjectMap[rr:predicate dc:description;
rr:predicate rdfs:comment;
rr:objectMap[rr:column "LABEL_CONF"; ];].
 
<#TripleMap2> a rr:TriplesMap ;
[rr:sqlQuery """ Select * from PREDICTIONS where LABEL =’Appealing’ """];
rr:subjectMap[rr:template "https://www.europeana.eu/en/item/{IMAGE_NAME}";
rr:predicateObjectMap[rr:predicate dc:subject;
rr:objectMap[rr:template  "http://purl.obolibrary.org/obo/MFOEM_000039";];].

An important aspect of the integration process involves the inclusion of certainty in the resulting semantic annotation. The area we are investigating involves a certain level of subjectivity. To represent the level of subjectivity in our semantic annotation, we add additional triples representing the annotation confidence as part of the description of the image, however, the representation of confidence/fuzzy knowledge needs to be addressed in the future.

Finally, the RDF data need to be integrated into the existing system. Although this is usually the task of the aggregators to decide on how to consume the annotation, our method is capable of generating the final data in a format specified by the user which includes RDF, TURTLE, NQUAD, or JSON-LD.

#### 3.3.2. Supporting Efficient Exploitation

This step focuses on the exploitation of semantic annotation by supporting efficient aggregation and exploration of the data. There are different ways of achieving this. First, by providing users new exploration paths (SPARQL Query Templates) to query the collections using the newly added ontology concepts and relationships as used in [45,46]. Second, by supporting visualisation of the collection using the new annotation as a criterion for aggregating images as in [47]. Third, the use of interactive chatbots that are trained based on the annotated data to support queries that are based on precompiled templates. Although the first two options can be implemented directly on existing semantic repositories, the last option requires further development of a chatbot that is trained on the data set [48,49].

## 4. Case Study

This case study is conducted in the framework of ChIA project (https://chia.acdh.oeaw.ac.at/ (accessed on 15 July 2021)) with a clear aim of engaging and testing new AI technologies against the background of a selected data set of food images for the benefit of accessing and analysing cultural data. The case study is applied following our proposed method (Section 3) which ensures the efficient representation of the data employing state-of-the-art semantic web technologies (ontologies and thesauri) and efficient analysis of the content using contemporary AI tools (CV). It presents a comprehensive methodology for answering how cultural knowledge of abstract food topics can be gained in a more structured and efficient method, and how this method is generalised to other areas in the digital humanities domain.

### 4.1. Phase-1: Preparation Phase

In the preparation phase, we identified potential platforms that contain huge collections of cultural images. The general Europeana platform partnering with Europeana-local Austria, for accessing the images and the infrastructure, is selected. Europeana Local is a network portal for local and regional cultural and scientific data. The focus of the Europeana Local project is the coordination and integration of the heterogeneous data sets at both national and local level. Furthermore, for supporting our endeavour, we focused on technologies that are related to AI, particularly related to CV and semantic web technology. We also considered semantic technologies that we would require to use along the several stages of the semantic enrichment process.

#### 4.1.1. Understanding and Defining the Domain

The subject of the images is restricted to the topic of food and drink. Some of the rationales for selecting the topic of food includes, first, the availability of large collections of images related to edible food from the Europeana database from which our project draws a considerable quantity of cultural heritage images representing a great variety of cultural content holders (such as museums, archives, libraries, botanic gardens) across Europe. Second, the topic of food is a common topic, that all people can relate to. This includes the kind of food we consume, how it is produced, the fashionable food—these facets are all closely related to our political and economic history. Third, there is a huge diversity of cultural information represented by food images. Even if defining a clear boundary of the food topic is difficult, we restricted the topic to the production, preparation, presentation, and consumption of edible food.

Three concepts and their complements were selected ranging from very objective and concrete objects (fruit/non-fruit) to abstract (formal/informal) to very abstract and subjective (appealing/non-appealing) concepts. The definitions for the respective image labels were composed of definitions available in monolingual dictionaries and encyclopedias, according to the best fit for the overall theme of the project. In this step, we used a web-based image annotation tool (MakeSense.AI (https://www.makesense.ai/ (accessed on 1 April 2021))) which provided the environment suitable for the tasks at hand. MakeSense.AI is a simple, freely available and customisable tool that made it suitable for annotating images by multiple annotators.

#### 4.1.2. Image Acquisition

**Figure 2:**
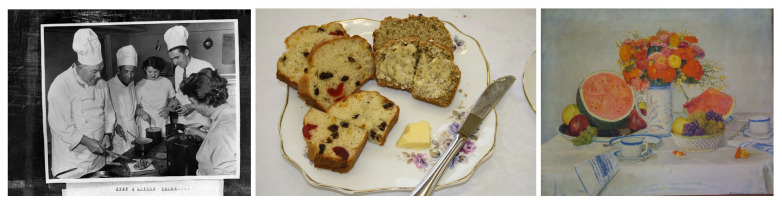
Sample food images selected from Europeana.

With the ChIA search platform established by Europeana-local Austria, we extracted food-related images (Refer Figure 2 for sample images) including paintings, photographs, and drawings. These images are extracted from the Europeana international platform that allows users to search images that contain food-related terminologies [50]. We collected more than 42,000 images in the first instance, grouped into several sub-folders representing the time, country, format, theme, etc. of the images. The search platform further allows the extraction of the digital images along with the associated metadata using RDF, XML or JSON-LD format generated for each image.

We further filtered images that are not related to food and drink. Since the initial selection of the sub-folders is based on food-related terminologies, the precision of the search was low and resulted in images that are not related to food. For example, the search apple resulted in several images of the Apple company and images related to Adam and Eve (due to their association with the apple tree). Generic food image detection tools were employed to further filter out images that are not related to food and drink [51]. For the final selection, a manual inspection of candidate files was conducted by Europeana local-At experts.

#### 4.1.3. Ontology Selection

To represent the concepts of food and drink efficiently, first we evaluated existing ontologies that are relevant to the topic of the research which focuses on ontologies related to food and drink as well as ontologies focusing on the cultural images. Finding ontologies that satisfy both requirements is difficult. Ontologies such as FoodOn (https://github.com/FoodOntology/foodon (accessed on 10 May 2021)), AGROVOC (http://www.fao.org/agrovoc/ (accessed on 12 May 2021)) [52] food ontology and others represent the topic of food and drink but lack the cultural representations, whereas ontologies such as Iconclass (http://www.iconclass.org/help/outline (accessed on 10 May 2021)) and Getty Art and Architecture Thesaurus (AAT) (https://www.getty.edu/research/tools/vocabularies/aat/index.html (accessed on 10 May 2021)) represent the cultural aspect along with some concepts related to food. Since any one of these ontologies does not fully satisfy our requirements (see Section 3.1.3, we created a vocabulary that maps existing and well-established food and art vocabularies to create an integrated food vocabulary focusing on food in cultural and historical imagery.

The two cultural ontologies selected, Iconclass and the Getty AAT, are both widely used vocabularies for describing image content in the arts. Iconclass was developed in the early 1950s by Henri van de Waal, professor of art history at Leiden University. Today, the thesaurus is maintained by the RKD Rijksbureau voor Kunsthistorische Documentatie (Dutch Institute for Art History). Iconography is the art and science of recording themes that frequently appear in works of art [53] and Iconclass is an iconographic classification system that offers a hierarchically organised set of concepts to describe the content of visual resources in representational Western art (ancient mythology and Christian religious iconography) [54].

The Getty AAT was created in 1980 and is supported by the Getty Art History Information Program since 1983 [55]. It is a large thesaurus that is continuously updated and currently comprises about 71,000 records and about 400,400 terms, including synonyms and related terms, relevant to the field of art (December 2020). The terms, descriptions, and other information for generic concepts concern art, architecture, conservation, archaeology, and other cultural heritage [56].

Some ontologies for food exist, but most of them have been developed for specific applications related to food and lack cultural aspects. Targeted ontologies have been developed for agriculture, certain popular products such as pizza (https://github.com/owlcs/pizza-ontology (accessed on 10 May 2021)) and wine (https://www.w3.org/TR/owl-guide/wine.rdf (accessed on 10 May 2021)), or in the context of culinary recipes, cooking, kitchen utensils, or nutrition. The FoodOn ontology was among the first attempts to build an ontology for broader applications. It includes nearly 30,000 terms about food and food-related human activities, such as agriculture, medicine, food safety control, shopping behaviour, and sustainable development [57].

In 2019, researchers created the FoodOntoMap [58] resource with the support of the Slovenian Research Agency programme and the H2020 project SAAM. FoodOntoMap consists of food concepts extracted from recipes, and thus foods that are edible for humans, and for each food concept, semantic tags from four food ontologies were assigned. The four ontologies used for matching were the Hansard corpus (https://www.english-corpora.org/hansard/ (accessed on 14 May 2021)), the FoodOn, OntoFood (https://bioportal.bioontology.org/ontologies/OF/?p=summary (accessed on 12 May 2021)) and SNOMED CT food (https://confluence.ihtsdotools.org/display/DOCEG (accessed on 14 May 2021)) ontologies. FoodOn is very comprehensive, and also provides semantics for food safety, food security, agricultural and animal husbandry practices associated with food production, culinary, nutritional and chemical ingredients and processes. As we only needed a selection of FoodOn concepts (human edible foods) for ChIA, FoodOntoMap offered us a perfect baseline for the ChIA vocabulary.

FoodOnToMap also provided us with an excellent base of matching concepts and we used this resource to update and expand with exact and related matches to the Iconclass and AAT ontology. Our goal was to add equivalence relationships between concepts that occur in the selected different ontologies and refer to the same entity in the world. The matching results from FoodOntoMap to AAT and Iconclass provided us with the first version of an integrated vocabulary of culture-related food terms with 1003 concepts, 1508 exact, and 1543 related matches from all processed food and art ontologies.

The resulting vocabulary is available at (http://chia.ait.co.at/vocab/ChIA/index.php, (accessed on 12 May 2021)) which provides details of food-related concepts and cultural concepts merged to represent cultural and historical food and drink-related concepts. Finally, the integrated ChIA food vocabulary was very well suited to search the Europeana corpus for food-related images and thus facilitated the repeated creation of training sets for data annotation.

### 4.2. Phase-2

#### 4.2.1. Analysis of the Contents of the Images

Due to the diversity and richness of the format and contents of the images, we conducted this phase in several iterations which we represented as rounds (Round-1, Round-2, Round-3, and Round-4). Each round served as a pilot study to determine the complexity of analysing the content of the images and generating high-quality annotation data. In each round, we executed different tasks (Task-1, Task-2, Task-3). These tasks represent only a fraction of potential concepts one can identify in the collection.

Task-1: involves the use of concrete food-related concepts. For the experiment, we selected a concrete concept “Fruit” and analysed the images by considering the presence/absence of fruit in the image. Fruit is selected due to its wider presence in the image collection and the concrete nature that makes it easier to be identified both by humans and existing CV tools with higher accuracy.

Task-2: focuses on images that contain abstract and subtle concepts that represent rich cultural aspects. In this task, we selected “Formal” and “Informal” concept categories and analysed the images by considering the setting where the food is presented.

Task-3: also focuses on the abstract and subjective concept that deals with appealing and non-appealing image categories. Definitions for images categorized as “Appealing” or “Non-appealing” depend on the overall aim of the project, after careful consultation of possible word definitions from online monolingual English dictionaries, such as Collins English Dictionary online, the American Heritage Dictionary and a book called “The Art Instinct,”. In this respect, in our project we define an image that is “Appealing” as “an image that is a pleasure to look at”; an image that is “Non-appealing” is “an image that is not a pleasure to look at”. We are aware that these are highly subjective and will vary depending on other parameters such as cultural background, food preferences, etc.

The abstract categories, unlike the concrete concepts, embed some level of abstraction that can not be formally detected by both humans and computers due to a high level of subjectivity based on culture, dietary style, geographic location, and other states of the annotators.

This case study particularly focused on Appealing and Non-appealing images. We believe that the use of the Appealing/Non-appealing concept represents the majority of the desired semantic enrichment of cultural images due to the following reasons. First, appealingness can be defined based on the features of the images including colour, brightness, orientation, etc. [59]. Second, appealingness is subjective and it varies from one society to another society, in the time horizon and based on the dietary preference of individuals. This makes the concept very representative of the topics of cultural images. Since modern AI technologies are applied for cultural images, we wanted to explore how a CV model would understand an image that represents abstract concepts.

#### 4.2.2. Manual Annotation for Generating Training Data

During the preparation of the training data, selecting the initial sets of images for the semantic annotation process and generating high-quality data for training a CV system was crucial. The initial candidate images and the subsequent images used in the manual annotation are selected by the Europeana-local Austria experts by evaluating the appropriateness of the images for the task. During each round of tasks, new images that were not used in the previous rounds were added.

Five annotators were involved throughout the process and some additional annotators are introduced for further validation of the annotation results. The five annotators came from different educational backgrounds (digital humanist, semantic web expert, computer scientist, CV expert, student, and socio-linguist), geographic locations (Europe, South America, Africa, and Asia), gender (two female and three male) and dietary preference (vegans and vegetarians included). Although the diversity of the annotators is a certain factor, we did not make any scientific selection of these annotators to base any further analysis on the effect of their background on the annotation results. Table 2 presents the Kappa agreement between five annotators in Round-1and Round-2 annotations. The results in the left column represent Kappa agreements from Round-1 where the annotators completed the tasks without consulting a formal definition of the annotation labels. The results in the right column present the Kappa agreements after the annotators are provided with a formal definition of all the categories. The effect of the presence of the formal definition can be compared in detail between Round-1 and Round-2 Kappa agreements for all three tasks.

#### 4.2.3. Round-1

The first round image selection resulted in identifying 392 cultural images. The images were annotated by five annotators using Fruit/Non-fruit, Formal/Informal, and Appealing/Non-appealing categories. Annotators were asked to annotate the images without consulting any formal definitions of the categories. The resulting annotations were analysed and an inter-annotator agreement was generated. A higher level of kappa agreement (0.928) was achieved for Fruit/Non-fruit category and a fair agreement (0.317) was achieved in the Formal/Informal category, whereas moderate (0.528) agreement was achieved in the appealing/non-appealing category (Refer Table 2).

#### 4.2.4. Round-2

In the second round, we formally defined all the concepts to investigate the effect of having common semantics on the inter-annotator agreement. The definitions (refer Table 3) were provided to the annotators before they started Round-2 annotation.

The effect of the formal definition for concrete concepts (Fruit/Non-fruit) demonstrated little impact (0.928 → 0.922) as the concepts were clear and straightforward. However, the use of formal description of the abstract concepts showed a slight improvement in the inter-annotator agreement by providing more clarity about the features the annotators should consider during the annotation and enabled them to be more self-consistent Formal/Informal (0.31 → 0.37) and Appealing/Non-appealing (0.52 → 0.50) (note the decrease for Appealing/Non-appealing category). The results of the inter-annotator agreements are presented in Table 2. The definition for the Appealing/Non-appealing category was adopted in Round-3 and Round-4.

#### 4.2.5. Round-3

Based on the lesson learned in Round-2, we focused only on the Appealing/Non-appealing category. We dropped Fruit/Non-fruit category because existing CV tools have already achieved a higher level of accuracy in identifying fruits in digital images [51,60,61]. We further dropped the Formal/Informal category as it resulted in a lower agreement. We took the Appealing/Non-appealing category as it represents an interesting aspect of cultural images. In this round, all the annotations from all three rounds were used to train a CV model.

From our image collection, we selected 1010 additional images and included them in the annotation process. Of all the six annotators who participated in this round, five annotators were familiar with the process and the sixth annotator was included upon getting familiar with the process. The inter-annotator agreement between the annotators is presented in Table 4. Apart from the kappa agreement, we generated the number of images classified as appealing and non-appealing using majority voting methods. In this round, we further build a CNN classifier using the collected annotation data as a data set. We used 830 images with greater than or equal to 66.7% vote. The annotation data was split into training, validation, and test sets. We trained three CNN models and identified the best performing model in Table 5. The Kappa agreement presented in the table shows that there is a fair agreement between the annotators on Round-3 images. The resulting models showed some promising results, however, the use of 830 images for training a model is not sufficient to make a reasonable conclusion. Another concern of using this data set as a basis for training a model was the difference between the number of Appealing and Non-appealing images, such imbalance created a bias in the CNN model, and hence to address this problem we selected additional 1079 images to run through Round-4 annotation.

#### 4.2.6. Round-4

Round-4 annotation aimed to increase the number of images for training and to balance the training data for the two categories (Appealing and Non-appealing). To achieve a high quality of the data set compared to the previous annotation round, the threshold was set to 80%. To achieve this, another 1079 images were added and the inter-annotator agreement is provided in Table 6. To build a balanced data set we reduced the number of images belonging to the Appealing category.

Once again, deep learning models were trained using 1010 images of which 545 images belonged to the Appealing category and 465 images belonged to the Non-appealing category. This image data was further divided into training, validation, and test set. There was an improvement in the distribution of images belonging to both categories in the final data. In combination with previously trained models on a huge data set, our image collection provides a very good set of training data with a reasonable annotation accuracy. The obtained data shows that compared to the human annotation, the accuracy of the CV models is superior in that, first, it always generates a consistent label (we observed that human annotators were not always consistent between the rounds), and second, it can be trained using active learning where the models learn by incorporating feedback from users.

In this round, we decided to include a confidence score of the CV models regarding the predicted category of a target image. This is a significant step that enables us to incorporate subjectivity as views related to a given culture are subjective. Detailed results of the CV models implemented are provided in Table 7. The best performing model in our case is the Fine-tuned Xception model which outperformed the other two models.

Although the four rounds enabled us to raise the quality and quantity of the training data sets, CV models benefit from large training data sets. As we demonstrated in our experiment, generating a large and high-quality data set in the cultural domain requires a huge resource, particularly the availability of expert annotators. However, our approach provides a methodology that ensures the incremental generation of high-quality training data set for domains with low resources.

### 4.3. Phase-4: Integration and Exploitation Phase

An important aspect of semantic annotation is the integration of the resulting annotation into an existing repository in a form that is suitable for both humans and machines to understand and interpret. The integration process first converts the resulting semantic annotation into s-p-o triples as discussed in Section 3.3.1. Second, integrating the newly generated triples into the existing semantic repository, and third, supporting efficient exploration of the data and exploitation of the images by the end-users. Each step is discussed as follows.

#### 4.3.1. Moving towards Large Scale Annotation

**Figure 3:**
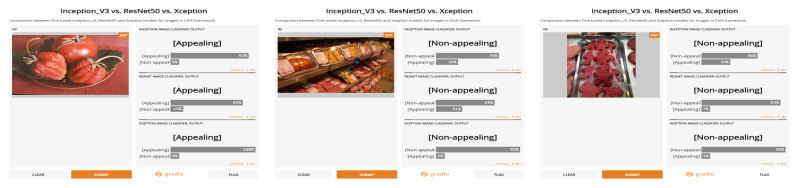

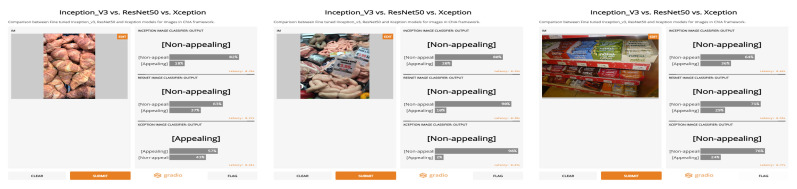
Sample images and their predicted categories using the three models. The images are cultural food images that are taken from the Europeana platform. The predictions of the three models indicate the categories (Appealing/Non-appealing) of the images along with the confidence score of each model.

The next step is the application of the trained models to predict the labels for the new and unseen images. At this stage, we have identified and trained three models with 81.68% to 95.98% training accuracy, and 84.5% to 88.5% validation accuracy. An image is classified and annotated with a particular class label by considering the average of the confidence level of the majority voted class, a similar approach has been followed in [62]. The prediction of some selected images using the three models along with the confidence scores is presented in Figure 3.

#### 4.3.2. Integration of Results

The annotations and their respective confidence scores are used to create s-p-o triples. These triples are generated for every image and the resulting data can be integrated into existing platforms following the preference of the aggregators. These data sets can be pushed to any triple store (including Europeana-local Austria) once verified by the aggregators. In this semantic interlinking stage, we link the images with concepts drawn from an ontology related to emotion (http://www.ontobee.org/ontology/MFOEM (accessed on 15 May 2021)) using a rdfs:type property. The images are also labelled as rdfs:type edm:WebResource. We further add the labels as part of the metadata using dc:description and rdfs:comment to represent it as a free-text account of the image resources. It is also observed that without a specialised ontology, the accurate interlinking of the annotation data with existing ontologies will not fully meet the objectives. Aggregators can link the target images using relationships and concepts by including additional triples using the R2RML mapping discussed in Section 3.3.1. For brevity, we present a snippet of the generated triples below.


<https://www.europeana.eu/en/item/2059511/data_foodanddrink_24255>  a

<http://www.europeana.eu/schemas/edm/webResource> ;

<http://www.w3.org/2000/01/rdf-schema#comment>  "Appealing" , "Appealing:95.69" ;

<http://purl.org/dc/elements/1.1/description>  "Appealing" , "Appealing:95.69" ;

<http://purl.org/dc/elements/1.1/subject>    <http://purl.obolibrary.org/obo/MFOEM_000039> .

<https://www.europeana.eu/en/item/2059511/data_foodanddrink_24264> a

<http://www.europeana.eu/schemas/edm/webResource> ;

<http://www.w3.org/2000/01/rdf-schema#comment>  "Appealing" , "Appealing:96.33" ;

<http://purl.org/dc/elements/1.1/description>  "Appealing" , "Appealing:96.33" ;

<http://purl.org/dc/elements/1.1/subject>   <http://purl.obolibrary.org/obo/MFOEM_000039> .

<https://www.europeana.eu/en/item/2059511/data_foodanddrink_24245>  a

<http://www.europeana.eu/schemas/edm/webResource> ;

<http://www.w3.org/2000/01/rdf-schema#comment>   "Non-appealing" , "Non-appealing:81.24" ;

<http://purl.org/dc/elements/1.1/description>  "Non-appealing" , "Non-appealing:81.24" .

<https://www.europeana.eu/en/item/2059511/data_foodanddrink_24263> a

<http://www.europeana.eu/schemas/edm/webResource> ;

<http://www.w3.org/2000/01/rdf-schema#comment>   "Appealing" , "Appealing:91.9" ;

<http://purl.org/dc/elements/1.1/description>  "Appealing" , "Appealing:91.9" ;

<http://purl.org/dc/elements/1.1/subject>  <http://purl.obolibrary.org/obo/MFOEM_000039> .


#### 4.3.3. Supporting Efficient Exploration

The methodology further provided mechanisms for efficient exploration of the resources by enabling exploration paths and templates. The following SPARQL templates are introduced to support the explorations [47]. The exploration of the triples is not restricted to the exploration paths, however becomes open and can be used for interlinking images with selected abstract cultural queries. Sample SPARQL query for extracting Appealing (Aesthetically Pleasing) images by filtering the subject using rdf:type and dc:subject predicates is shown below.

prefix obo: <http://purl.obolibrary.org/obo/> prefix rdf: <http://www.w3.org/1999/02/22-rdf-syntax-ns#> prefix rdfs: <http://www.w3.org/2000/01/rdf-schema#> prefix dc: <http://purl.org/dc/elements/1.1/> prefix edm: <http://www.europeana.eu/schemas/edm/>
 
select ?subject ?predicate ?object
where{
?subject ?predicate ?object.
?subject rdf:type edm:webResource.
?subject dc:subject obo:MFOEM_000039.
}
limit 15

A snippet of the output of the above query is given below. The result shows the potential of the newly generated metadata to enrich, interlink and group images by tagging them using one or more abstract cultural heritage concepts.


<https://www.europeana.eu/en/item/2059511/data_foodanddrink_24255> dc:subject  obo:MFOEM_000039

<https://www.europeana.eu/en/item/2059511/data_foodanddrink_24255> dc:description  Appealing

<https://www.europeana.eu/en/item/2059511/data_foodanddrink_24255> dc:description  Appealing:95.69

<https://www.europeana.eu/en/item/2059511/data_foodanddrink_24255> rdfs:comment  Appealing

<https://www.europeana.eu/en/item/2059511/data_foodanddrink_24255> rdfs:comment  Appealing:95.69

<https://www.europeana.eu/en/item/2059511/data_foodanddrink_24255> rdf:type  edm:webResource

<https://www.europeana.eu/en/item/2059511/data_foodanddrink_24264> dc:subject  obo:MFOEM_000039

<https://www.europeana.eu/en/item/2059511/data_foodanddrink_24264> dc:description  Appealing

<https://www.europeana.eu/en/item/2059511/data_foodanddrink_24264> dc:description  Appealing:96.33

<https://www.europeana.eu/en/item/2059511/data_foodanddrink_24264> rdfs:comment  Appealing

<https://www.europeana.eu/en/item/2059511/data_foodanddrink_24264> rdfs:comment  Appealing:96.33

<https://www.europeana.eu/en/item/2059511/data_foodanddrink_24264> rdf:type  edm:webResource

<https://www.europeana.eu/en/item/2059511/data_foodanddrink_24263> dc:subject  obo:MFOEM_000039

<https://www.europeana.eu/en/item/2059511/data_foodanddrink_24263> dc:description  Appealing

<https://www.europeana.eu/en/item/2059511/data_foodanddrink_24263> dc:description  Appealing:91.9


The application of our method to represent and annotate cultural images using abstract concepts is scalable when additional cultural concepts are used to annotate the target images. Depending on the requirements, it supports the extraction of images linked to external repositories.

## 5. Discussion

Even if the individual phases and steps proposed in our methodology are not new, this paper presents a novel and efficient combination of the steps that fit the purpose. The application of the methodology in the case study on Europeana cultural heritage images exposed the problems and the challenges not only from the methodological but also technical and practical perspectives. This paper implements the methodology, first by applying ontologies to consistently represent concepts, second, using CV tools to enrich cultural images by training models that can be applied to large data sets, third, by enabling existing systems to efficiently support user requirements, and finally integrating subjectivity and fuzziness into the metadata. Lessons learned from each of the contributions are summarised below.

Firstly, although several ontologies and vocabularies are available, the selection and composition of ontologies that represent the knowledge base of cultural and historical aspects are not fully explored. The composition of cultural heritage concepts from existing generic or specific ontologies is difficult and deserves a proper investigation. It is evident from our case study that several cultural aspects (including family status, economic status, style, nutrition, etc.) are embedded in the images which need to be formally defined using vocabularies. As an example, we searched Linked Open Vocabulary (LOV (https://lov.linkeddata.es/dataset/lov/ (accessed on 15 July 2021))) repository for concepts representing appealingness and attractiveness defined concerning food images. Our search resulted in a few concepts that are only related to computer software quality features. All these aspects can not be covered in a single ontology, therefore the integration of several existing ontologies and the development of new ones is crucial. In addition to the vocabularies, domain-specific relationships between the images and the concepts need to be defined. We came across cases where the annotated features of the images can not be embedded using existing generic relations. In this regard, ontologies play a major role in defining rich relationships (owl:ObjectProperty) between cultural concepts.

Secondly, CV tools have provided significant breakthroughs in detecting objects. Although they lag in identifying exceptional cases. Our case study demonstrates that they can be effectively exploited. The case study expanded the state of the art by including the detection of abstract concepts that are very subjective and difficult to quantify. We are aware that there are significant omissions of exceptional cases by the AI and ML algorithms [63] and tried to reduce the bias by incorporating confidence scores. We are also aware that the kappa inter-annotator agreement may cause an issue in the interpretation of the agreements as poor, slight, fair, moderate, substantial, and strong [64,65]. One limitation of our method is that the use of Kappa agreement and its interpretation which may not be suitable for mission-critical tasks such as in medical applications [66,67]. To reduce the effect of outlier cases, we embedded the confidence levels of the predicted annotations. After all, for such abstract concepts, the experiment also showed that the inter-annotator agreements are low or moderate compared to that of concrete concepts. Round-2 experiment enabled us to identify some interesting aspects of cultural image enrichment. Concrete concepts can be annotated by existing image recognition tools with high accuracy [68], whereas abstract concepts are fuzzy even when the annotation is done by human experts.

Thirdly, the digital humanities domain, particularly cultural heritage could benefit from existing semantic web and AI domains in several ways. However, our experiment also showed that there is a lot of work that needs to be done to ensure the quality during the digitization process of cultural heritage resources. The integration of new annotations on top of existing annotations should not introduce an inconsistent interpretation of the target resources.

Finally, our research contributes additional data sets to the research community. The data set includes more than 2000 images that are annotated by five annotators. This data set can be used as a benchmark for evaluating future models and also serve as a starting point for future crowd annotation. Our food vocabulary is another contribution to the domain in that it amalgamates food concepts from different sources into one.

## 6. Conclusions

We presented a methodology for semantic enrichment of digital cultural heritage images covering the domain of cultural food images. A three-phase methodology is proposed and a case study following the methodology is implemented in the context of a 2-year ChIA project. The proposed methodology provides a structured approach that enables digital humanities experts to identify, enrich and publish their cultural heritage-related collections using LOD formats. It also provides guidance and directions on how existing artificial intelligence tools such as CV and semantic web technologies can be combined and exploited efficiently in the digital humanities domain. This paper explored the introduction of abstract and subjective concepts into the proposed semantic enrichment process and identified the challenges and opportunities that exist in the emerging AI-based technologies. We believe that the confidence of the CV model can be continually improved by incorporating active learning steps in the methodology. In future work, we will integrate an active learning component, as discussed in [15], where users will be provided options to rate the output while the model continually improves its performance by learning from the feedback. Another future research will be contributing towards an ontology of abstract cultural concepts and the preparation of high-quality data sets that can be used in similar research settings.

## Figures and Tables

**Table 1:** Expected annotation of cultural heritage images with confidence.

Image_Name	Label	Confidence
https://image1	Concept 1	85%
https://image1	Concept 2	100%
https://image1	Concept 3	40%
...	...	...

**Table 2:** Inter-annotator agreement for Round-1 and Round-2 annotation.

Round-1						Round-2					
	**A001**	**A002**	**A003**	**A004**	**A005**		**A001**	**A002**	**A004**	**A005**	**A006**
Task-1: Fruit/Non-fruit.											
A001	1.000	0.928	0.892	0.907	0.886	A001	1.000	0.943	0.928	0.913	0.912
A002	0.928	1.000	0.892	0.938	0.897	A002	0.943	1.000	0.912	0.866	0.865
A003	0.892	0.892	1.000	0.923	0.923	A004	0.928	0.912	1.000	0.913	0.881
A004	0.907	0.938	0.923	1.000	0.918	A005	0.913	0.866	0.913	1.000	0.897
A005	0.886	0.897	0.923	0.918	1.000	A006	0.912	0.865	0.881	0.897	1.000
Task-2: Formal/Informal.											
A001	1.000	0.330	0.252	0.316	−0.091	A001	1.000	0.168	0.255	0.167	0.125
A002	0.330	1.000	0.210	0.306	0.153	A002	0.168	1.000	0.095	0.419	0.489
A003	0.252	0.210	1.000	0.051	−0.031	A004	0.255	0.095	1.000	0.089	0.208
A004	0.316	0.306	0.051	1.000	−0.028	A005	0.167	0.419	0.089	1.000	0.520
A005	−0.091	0.153	−0.031	−0.028	1.000	A006	0.125	0.489	0.208	0.520	1.000
Task-3: Appealing/Non-appealing.											
A001	1.000	0.659	0.296	0.534	0.317	A001	1.000	0.472	0.526	0.336	0.569
A002	0.659	1.000	0.325	0.453	0.268	A002	0.472	1.000	0.565	0.454	0.366
A003	0.296	0.325	1.000	0.424	0.370	A004	0.526	0.565	1.000	0.439	0.386
A004	0.534	0.453	0.424	1.000	0.454	A005	0.336	0.454	0.439	1.000	0.205
A005	0.317	0.268	0.370	0.454	1.000	A006	0.569	0.366	0.386	0.205	1.000

**Table 3:** The definitions used for Round-2 ChIA image classification.

Concept	Definition
Fruit/Non-fruit	Fruit: a fruit is something that grows on a tree or bush and which contains seeds or a stone covered by a substance that you can eat. (e.g., strawberry, nut, tomato, peach, banana, green beans, melon, apple). Non-fruit: images that do not feature any type of fruit (for fruit definition see above)
Formal/Informal	Formal: arranged in a very controlled way or according to certain rules; an official situation or context. Informal: a relaxed environment, an unofficial situation or context, disorderly arrangement.
Appealing/Non-appealing	Appealing: an image that is a pleasure to look at. A food image that is pleasing to the eye, desirable to eat and good for food. Non-appealing: an image that is not a pleasure to look at.

**Table 4:** Inter-annotator agreement for Round-3 annotation of 1010 images (Appealing/Non-Appealing).

	A001	A002	A004	A005	A006	A007
A001	1.000	0.293	0.335	0.330	0.164	0.274
A002	0.293	1.000	0.475	0.483	0.190	0.042
A004	0.335	0.475	1.000	0.648	0.156	0.082
A005	0.330	0.483	0.648	1.000	0.123	0.061
A006	0.164	0.190	0.156	0.123	1.000	−0.025
A007	0.274	0.042	0.082	0.061	−0.025	1.000

**Table 5:** Results of training deep learning models for image classification: Round-3.

Model	Training Accuracy	Validation Accuracy	Test Accuracy
Fine tuned ResNet50	83.51%	83.81%	80%
Fine tuned Inception_V3	92.61%	87.62%	90%
Fine tuned Xception	93.2%	88.1%	85.56%

**Table 6:** Inter-annotator agreement for Round-4 annotation of 1079 images (Appealing/Non-Appealing).

	A001	A002	A004	A005	A006
A001	1.000	0.223	0.287	0.057	0.293
A002	0.223	1.000	0.245	0.090	0.163
A004	0.287	0.245	1.000	0.133	0.200
A005	0.057	0.090	0.133	1.000	0.085
A006	0.293	0.163	0.200	0.085	1.000

**Table 7:** Results of training deep learning models for image classification: Round-4.

Model	Training Accuracy	Validation Accuracy	Test Accuracy
Fine tuned ResNet50	87.47%	84.5%	83.85%
Fine tuned Inception_V3	81.68%	85.5%	88.46%
Fine tuned Xception	95.98%	88.5%	90.77%

## Data Availability

The image data used in this research is available at the ChIA website (https://chia.acdh.oeaw.ac.at/data/ (accessed on 15 July 2021)). The data set includes the URLs to the images along with annotations from all rounds. data set predicting the labels for additional images is also included. The ChIA vocabulary is available at Europeana local-At (http://chia.ait.co.at/vocab/ChIA/index.php (accessed on 15 July 2021)). Finally, the three trained models are also available as IPython Notebooks (https://github.com/acdh-oeaw/Chia/blob/master/notebooks/nnetworks/ (accessed on 15 July 2021)).
